# Energy extraction from nuts: walnuts, almonds and pistachios

**DOI:** 10.1017/S0007114519002630

**Published:** 2019-10-17

**Authors:** B. M. McArthur, R. D. Mattes

**Affiliations:** 1 Department of Food Science, Purdue University, West Lafayette, IN 47906, USA; 2 Department of Nutrition Science, Purdue University, West Lafayette, IN 47906, USA

**Keywords:** Walnuts, Nuts, Digestion, Energy extraction, Lipid bioaccessibility

## Abstract

The bioaccessibility of fat has implications for satiety and postprandial lipidaemia. The prevailing view holds that the integrity of plant cell wall structure is the primary determinant of energy and nutrient extraction from plant cells as they pass through the gastrointestinal (GI) tract. However, comparisons across nuts (walnuts, almonds and pistachios) with varying physical properties do not support this view. In the present study, masticated samples of three nuts from healthy adults were exposed to a static model of gastric digestion followed by simulated intestinal digestion. Primary outcomes were particle size and lipid release at each phase of digestion. Walnuts produced a significantly larger particle size post-mastication compared with almonds. Under gastric and intestinal conditions, the particle size was larger for walnuts compared with pistachios and almonds (*P* < 0·05). However, the masticated and digesta particle sizes were not related to the integrity of cell walls or lipid release. The total lipid release was comparable between nuts after the *in vitro* intestinal phase (*P* > 0·05). Microstructural examination showed ruptured and fissured cell walls that would allow digestion of cellular contents, and this may be governed by internal cellular properties such as oil body state. Furthermore, the cell walls of walnuts tend to rupture rather than separate and as walnut tissue passes through the GI tract, lipids tend to coalesce reducing digestion efficiency.

Walnuts have high satiety value, evoke a low postprandial lipaemic response and protect against metabolic disorders such as CVD and type 2 diabetes^(1–7)^. Additionally, the energy they contain is not efficiently absorbed, accounting for the limited impact they have on energy balance. The low bioaccessibilty of lipid from walnuts, and other nuts, has been attributed primarily to the presence of intact cell walls that hinder access/binding of lipases to oil bodies (OB) enclosed within the cells^(8)^. Where cell structures remain intact, nutrients (e.g. lipids, protein and vitamin E) are lost via faecal excretion^(9–11)^. However, mechanical (e.g. chewing, chopping and grinding) or thermal degradation of cellular structures promotes the ingress of digestive enzymes and liberation of intracellular nutrients that are then digested^(12–14)^. When access is not limited, for example, as in isolated OB or finely ground nuts, structural features of lipid control the extent of lipolysis^(15)^.

Consistent with these physical properties, randomised controlled trials have shown decreases in postprandial TAG responses in humans fed muffins with whole nuts compared with milled nuts^(6,7)^ as well as improved accessibility of nutrients with decreased size of masticated almond particles^(12)^. However, these investigations have mostly concentrated on the effects of altering the form (e.g. whole, milled, homogenised and roasted) more than the type of nut. Indeed, human studies have reported that there are appreciable differences in the digestion and release of lipid from different nut types: pistachios > almonds ≈walnuts^(16–18)^. These findings do not coincide with predictions based on the physical properties (i.e. hardness) of these nuts. Likely, the effect of nut type on lipid digestibility relates to the way that nuts are degraded during transit through the gastrointestinal (GI) tract, but direct evidence is not available.

Mastication is a primary determinant of the bioaccessibilty of lipid (and other nutrients) and the subsequent postprandial responses. It therefore warrants consideration for its potential role in walnut lipid bioaccessiblity. Previous *in vivo* studies report that boluses formed from hard, brittle foods, such as almonds, consist of large particles that contain mostly intact cells with low lipid bioaccesssibility^(8,12)^. These and other studies showed that during mastication, some cell walls rupture and their contents become exposed to digestive enzymes. However, no studies have examined whether chewing has equivalent effects on less brittle nuts, such as walnuts. Plant foods with a soft texture generally separate rather than fracture under pressure, resulting in small intact particles during mastication. The maintenance of intact cell walls may reduce the release of nutrients in the digestive tract, as has been shown for fruits and vegetables^(19,20)^. Whether this finding holds for walnuts has not been studied and warrants investigation. Additionally, intensive thermal or mechanical processing conditions result in a loss of structural integrity which leads to more fractured cells during mastication and a higher accessibility/absorption of nutrients as shown for roasted compared with raw nuts^(21,22)^. Walnuts are most frequently consumed raw so should be less susceptible to this effect.

The aim of the present study was to provide insight into the structural and biochemical degradation of walnuts during mastication as well as simulated GI (gastric and intestinal) digestion and its effects on lipid release. Interest in this question was driven by reports that extraction of energy from almonds, walnuts and pistachios is approximately 20, 21 and 5 %, respectively^(16–18)^. These values do not coincide with physical properties (i.e. hardness). We hypothesised that walnuts, being less brittle, would be chewed into smaller particle sizes, but their cells would separate under applied force and therefore elicit a low bioaccessibility of energy comparable with almonds. Alternatively, a recent *in vitro* study showed that more than 90 % of nutrients from pistachios are released in the gastric compartment^(23)^. Hence, pistachios appear to be more structurally degraded during digestive transit and therefore would exhibit greater nutrient losses than walnuts and almonds.

## Materials and methods

### Materials

Whole nuts were used in the present study. The walnuts were unsalted and provided by the California Walnut Commission. The almonds were roasted and salted and were provided by the Almond Board of California. Pistachios were dry-roasted (Kraft Heinz Foods Company) and were purchased from a local retailer in West Lafayette, IN, USA. These forms were selected as they are the most commonly consumed forms. The nuts were stored in sealed containers at 4°C until the day of testing. Digestive enzymes, porcine pepsin (no. P-7125; ≤400 unit/mg powder), porcine pancreatin (no. P-1750; 4 × USP-US Pharmacopeia specification), lipase from porcine pancreas type II (no. L3126; 100–400 unit/mg powder) and bile extract porcine (EC 232-369-0) were purchased from Sigma-Aldrich. The same material lots were used for all digestion experiments. All other chemicals and solvents in the present study were of analytical grade.

#### *In vivo* mastication

Mastication of nuts for the *in vitro* experiments was conducted by seven healthy volunteers (age 28 (sd 4) years; BMI 25 (sd 1·19) kg/m^2^; sex: three males, four females) according to the procedure of Grundy *et al.* ^(12)^, with modifications in relation to the starting material. Sample size calculations, using G-Power 3.1.2, were based on four participants completing the study at 80 % power and an *α*-level of 0·05 to detect a 0·2 difference in the percentage of total lipid released with a sd of differences of 1 using data from a pilot study. On 2 separate testing days, volunteers reported to the laboratory where they were presented with four 5 g portions of nuts (walnuts, almonds and pistachios) in a random order. Volunteers were asked to chew each nut until they felt the urge to swallow, at which time they expectorated the sample into individual pre-weighted plastic (50 ml) centrifuge tubes. They then rinsed their mouth with 20 ml of water and emptied the rinse into the same tube to create a final volume of 30 ml. All expectorated boluses were used in the static *in vitro* digestion model, simulating gastric and intestinal digestion. Individual samples (1 ml) were taken immediately after the oral phase, at the end of the *in vitro* gastric digestion phase and at the end of the *in vitro* intestinal digestion phase and were stored at 4°C before particle size determination on the same day and for microscopy analysis. The present study was conducted according to the guidelines laid down in the Declaration of Helsinki, and the Purdue University Institute Review Board, USA, approved all procedures involving volunteers. The protocol number was 1504015989 and it was approved on 12 May 2015. Written informed consent was obtained from all volunteers.

#### *In vitro* gastrointestinal digestion

**Fig. 1.**
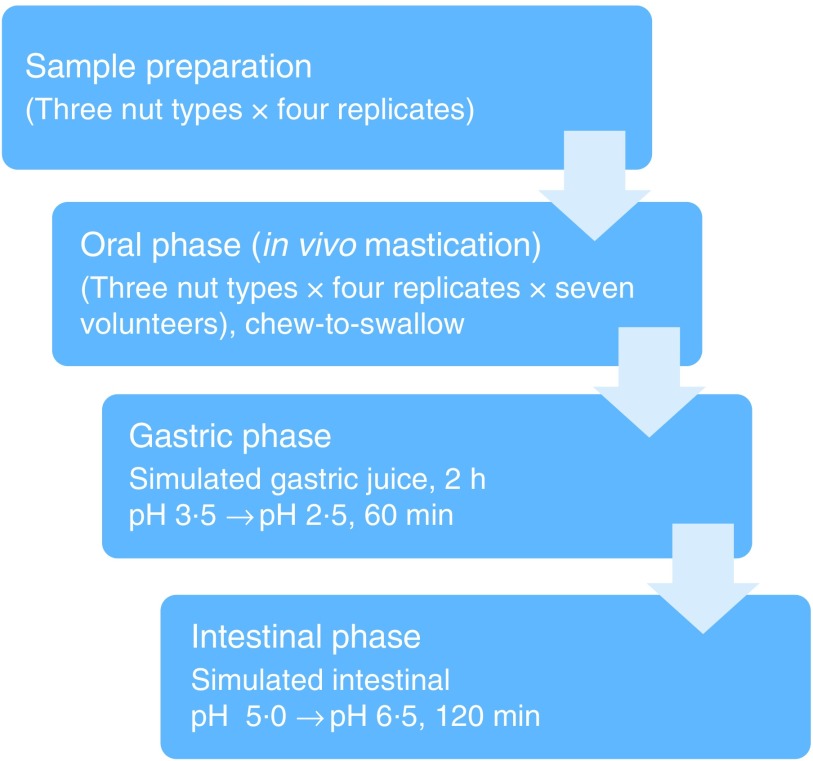
Overview of the digestion experimental procedures.

A flow diagram of the experimental procedure for the digestion model is shown in Fig. 1. *In vitro* digestions simulating gastric and intestinal digestion were performed as described by Lipkie *et al*.^(24)^. Gastric digestion was carried out immediately after the oral phase on the chewed nut samples. Samples (30 ml) were vortexed and acidified with 1·0 m HCl until it reached pH 3·5 ± 0·1. Then, gastric digestion was performed with the addition of 2 ml of pepsin solution (2000 U/ml), and the pH of the mixture was adjusted once more to 2·5 ± 0·1 with 1·0 m HCl. The final volume was adjusted to 40 ml with saline (0·9 % NaCl), capped with N_2_ to minimise contact with O_2_ and then incubated at 37°C in a shaking water bath for 60 min. Thereafter, the pH of the digesta was adjusted to 5·0 ± 0·1 with 1 m NaHCO_3_. The intestinal digestion was performed with the addition of 2 ml of pancreatin-lipase (2000 U/ml) solution and 3 ml of bile (10 mm). Further, the pH was adjusted to 6·5 ± 0·1 with 1 m NaHCO_3_, and the final volume was brought to 50 ml with saline, after which the headspace of the tube was flushed again with N_2_ and incubated in a shaking water bath at 37°C for 120 min. Following the intestinal digestion, the digesta was subjected to 60 min of 10 000 ***g*** centrifugation (Allegra X-22 R, Beckman Coulters) to remove the aqueous fraction and isolate the suspended particles. The recovered particles were washed with water and stored at 4°C for further experiments. All *in vitro* digestions were performed in quadruplicate. Samples and replicates were run in randomised order.

#### Particle size

The protocol used for the particle size measurements was adapted from the previous work^(12,25)^. An equal aliquot of sample that was collected after mastication and simulated gastric and intestinal digestion (walnuts, *n* 4; almonds, *n* 4; pistachios *n* 4) was poured onto a 2000 µm aperture sieve (WS Tyler) placed on top of a sieve base (36 µm mesh size) and then washed with 20 ml of deionised water. Once the water passed through the mesh, retained particles were transferred into a 1000 ml beaker. Particle sizes >2000 and <36 µm were removed to prevent obstruction in the instrument and interference with the measurements, respectively. Small particles (<36 µm) have been reported to correspond only to cell wall fragments and intracellular contents^(12)^. Suspended particles were loaded into a light scattering apparatus (Malvern Mastersizer HU 2000, Malvern Instruments Ltd). The refractive indices of the walnuts, almonds, pistachios and water are 1·47^(26)^, 1·46^(27)^, 1·46^(25)^ and 1·33, respectively. The speeds of the stirrer and the pump were 700 and 1175 rpm, respectively. Ten consecutive 10-s measurements were taken for each sample, to give the average particle size distribution of the digested nuts. The mean volume diameter (d_[4,3]_) of the particle was calculated from the intensity profile of the scattered light with the Mie theory by the use of the instrument’s software.

#### Total lipid extraction

Undigested nuts and digested residues, recovered at the end of the intestinal phase, were analysed for total lipid using a Soxhlet extraction method^(28)^, with petroleum ether as the solvent. The digested residues were centrifuged (2500 ***g***, 10 min) prior to analyses to remove the residual liquid phase. The residues were then dried and analysed. The results of lipid content analysis are expressed as a percentage of fresh weight. The relative bioaccessiblity of lipid in the nuts was calculated as follows (Eq. (1)):(1)
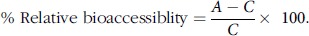
In this equation, A represents the lipid present in the original undigested sample; C represents the lipid retained in the digested material (non-bioaccessible fraction). A and C were calculated as a percentage of dry weight. In the present study, bioaccessiblity refers to the proportion of a lipid that is released from the food matrix and is potentially available for absorption in the small intestine.

#### Microscopy analysis

Microstructural analysis of undigested and digested nut cotyledon tissue was performed using light microscopy (LM) and transmission electron microscopy (TEM). Nut tissues were fixed with 2·25 % (v/v) glutaraldehyde in 0·1 m sodium cacodylate buffer (pH 7·4) and then post-fixed with a buffered 1 % osmium tetroxide solution containing 0·8 % potassium ferricyanide (pH 7·4) and left overnight. The specimens were then dehydrated in ethanol serial dilutions: 50, 70 and 95 % (v/v) ethanol in distilled water for 10 min intervals and then finally for three 10 min intervals in 100 % (v/v) ethanol^(29)^. For LM and TEM, specimens were embedded in Embed 812 resin and placed in moulds and polymerised at 70°C. Semi-thick sections (0·5 μm) for LM were cut on a Reichert-Jung Ultracut E ultramicrotome (Leica Microsystem Ltd), mounted on a glass slide and then stained with 1 % (v/w) toluidine blue. Specimens were then immediately viewed with a light microscope (Leica System Microscope, W. Nuhsbaum, Inc.) with LAS V4.3 software. Thin sections (80 nm) for TEM were cut on the same microtome and stained in 2 % uranyl acetate (w/v) and lead citrate. Specimens examined by TEM were viewed on a FEI Tecnai G^2^T12 transmission electron microscope (FEI Europe) equipped with a tungsten source and operating at 80 kV.

**Fig. 2.**
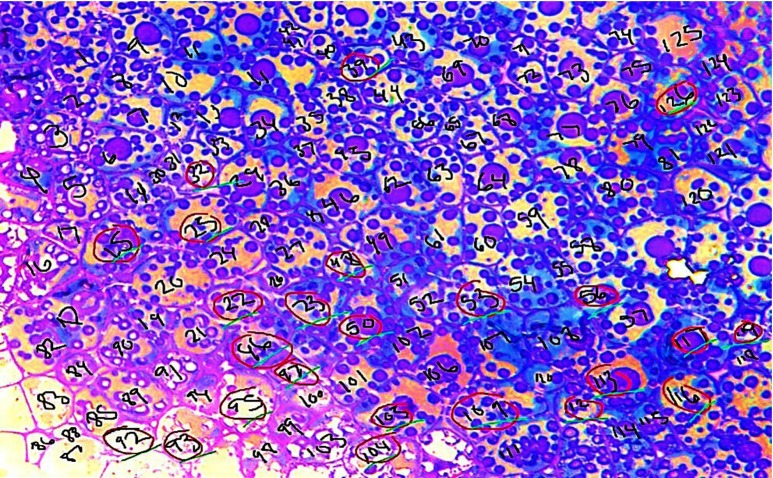
Light microscopy image of chewed almonds; note randomly selected areas within the parenchyma tissue of a nut were used to quantify the proportion of ruptured (circled) cells. In this figure, there are 126 cells total and an estimated twenty-two cells are ruptured. Hence, the proportion of ruptured cells is 17 % (i.e. 22/126 = 0·17 × 100 = 17 %).

The quantitative measurements of parenchymal cells were made using the images acquired from LM and TEM. To provide information about the structural integrity of cell walls, the proportion of ruptured to intact cells following mastication and *in vitro* digestion was estimated using the LM micrographs. In the present study, cells with a visible fissure were defined as ruptured cells. After each stage of digestion, LM was captured (40×) from three randomly selected areas within the cotyledon tissue of each nut. The three selected areas contained 100 cells. The total number of cells examined per nut was as follows: 3 digestion phase × 1 nut/digestion phase × 100 cells/nut = 300 cells per nut. The number of ruptured cells in the whole area of the micrograph was manually counted; results were expressed as a percentage of the total number of cells. A sample calculation is shown in Fig. 2.

**Fig. 3.**
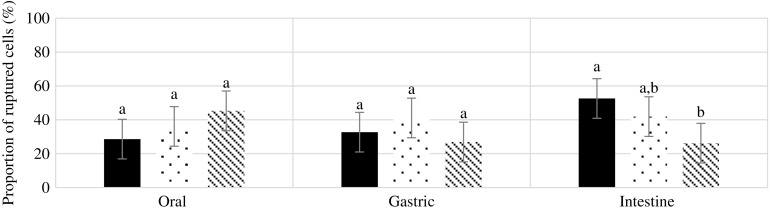
Mean proportion of ruptured cells for different nuts after each phase of digestion. Values are mean values of four replicates, with their standard errors represented by vertical bars. ^a,b^ Mean values with unlike letters are significantly different (*P* < 0·05; repeated-measures ANOVA). 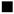, Walnuts; 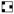, Almonds; 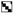, Pistachios.

Image analysis (ImageJ software, NIH) of TEM micrographs was used to quantify the thickness of cell walls, diameter of OB and cell size. For measurements of cell wall thickness/lipids, ImageJ’s ‘line selection’ and ‘measure’ (Analyse-Measure) tools were used for each cell on the original image. Cell size was calculated according to the method described by Grassby *et al*.^(30)^ (Eq. (2)):(2)
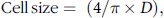
where *D* is the actual measured diameter of the cell.

### Statistical analysis

Data were analysed using SPSS version 22.0 (IBM Corp.). Statistical significance was set at a probability level of 0·05 (*P* < 0·05). All data were normally distributed (analysed by using the Shapiro–Wilk test). Linear mixed models with repeated measures were used to test for differences in particle size, lipid release, integrity of cell walls, dimensions of cells and OB. Nut type, replicate and digestion phase were treated as fixed effects. *Post hoc* analysis using Bonferroni adjustments was applied to examine pairwise differences. Results are expressed as mean values with their standard errors.

## Results

### *In vivo* mastication

The number of chews was statistically different between nuts (*P* < 0·05). More chewing cycles were necessary to reach swallowing for the almonds than the walnuts and pistachios (*P* = 0·01), but no differences were observed between the walnuts and pistachios (*P* > 0·05). The average number of chews per nut was 35 ± 4 for the almonds (mean values ranged from 20 to 60), 30 ± 4 for the walnuts (16–53) and 30 ± 4 for the pistachios (14–55).

### Particle size distribution

Table 1 presents the mean particle size (d_[4,3]_) of the different nuts after the three phases of digestion. The phase of digestion had a significant effect on the particle size of the walnuts and pistachios (both *P* < 0·005), but the particle size did not differ significantly across phases for the almonds. Walnuts produced particles that were significantly larger post-intestinal digestion (396 ± 10 µm) than oral (338 ± 10 µm) and gastric digestion (340 ± 10 µm). Similarly, pistachio particles were larger after the intestinal phase (347 ± 10 µm) compared with the oral (317 ± 10 µm) (*P* = 0·004) and gastric phases (290 ± 10 µm) (*P* < 0·005). Moreover, there is an effect of nut type on the mean particle size following digestion (Table 2). The mean particle size was significantly larger for the walnuts than the almonds following oral digestion (*P* = 0·010), but not different than the pistachios (*P* = 0·084). No significant differences between the pistachios and almonds were observed post oral digestion. The average particle size was significantly larger for the walnuts after gastric digestion (340 ± 10) compared with the almonds (292 ± 10) and pistachios (290 ± 10) (both *P* < 0·005). Following intestinal digestion, the mean particle size was larger for the walnuts (396 ± 10 µm) than the almonds (301 ± 10 µm) and pistachios (347 ± 10 µm) (both *P* < 0·005). Almonds yielded the smallest particle size after intestinal digestion (*P* < 0·0005).

**Table 1.** Mean particle size comparisons between digestion phases for each nut (Mean values with their standard errors; *n* 7)

	Walnuts		Almonds		Pistachios	
	Mean	sem	Mean	sem	Mean	sem
Oral (µm)	338^a^ (mean values range from 199·29 to 419·07)	10	308^b^ (mean values range from 197·71 to 404·71)	10	316^a^ (mean values range from 330·34 to 465·40)	10
Gastric (µm)	340^a^ (mean values range from 235·55 to 396·02)	10	292^b^ (mean values range from 239·04 to 337·68)	10	290^b^ (mean values range from 216·66 to 417·79)	10
Intestine (µm)	396^a^ (mean values range from 254·05 to 369·27)	10	301^b^ (mean values range from 160·92 to 355·58)	10	347^c^ (mean values range from 294·74 to 439·68)	10

a,b,c Mean values in a column with unlike superscript letters are significantly different (*P* < 0·05; repeated-measures ANOVA).

**Table 2.** Mean particle size comparisons between nuts after each digestion phase (Mean values with their standard errors; *n* 7)

	Oral		Gastric		Intestine	
	Mean	sem	Mean	sem	Mean	sem
Walnuts (µm)	338^a^	10	340^a^	10	396^a^	10
Almonds (µm)	308^a^	10	292^a^	10	301^b^	10
Pistachio (µm)	316^c^	10	290^a^	10	347^c^	10

a,b,c Mean values in a column with unlike superscript letters are significantly different (*P* < 0·05; repeated-measures ANOVA).

### Proportion of ruptured cells

There was no main effect of nut type or digestion phase on cell wall rupturing (*P* > 0·05) (Fig. 3). However, there was a significant interaction (*P* = 0·007). Pairwise comparisons showed that significantly more walnut cells were ruptured after the intestinal phase compared with pistachio cells (*P* = 0·005).

### Lipid bioaccessibility

The mean total lipid content present in the undigested walnuts, almonds and pistachios was 66, 50 and 46 % w/w, respectively. These values are similar to those found in the literature. Approximately 77, 76 and 78 % of the original lipid in the walnuts, almonds and pistachios, respectively, were released following the intestinal phase of digestion, with no significant differences between these nuts (*P* > 0·05).

### Microstructure

**Fig. 4.**
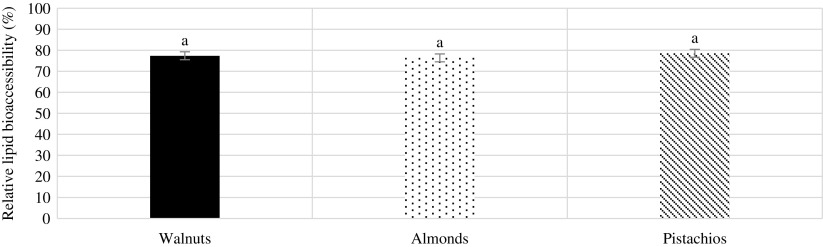
Relative lipid bioaccessibility as a percentage of the total lipid. Values are mean values of four replicates, with their standard errors represented by vertical bars. ^a^ Mean values with the same letter are not significantly different (*P* > 0·05; repeated-measures ANOVA).

**Fig. 5.**
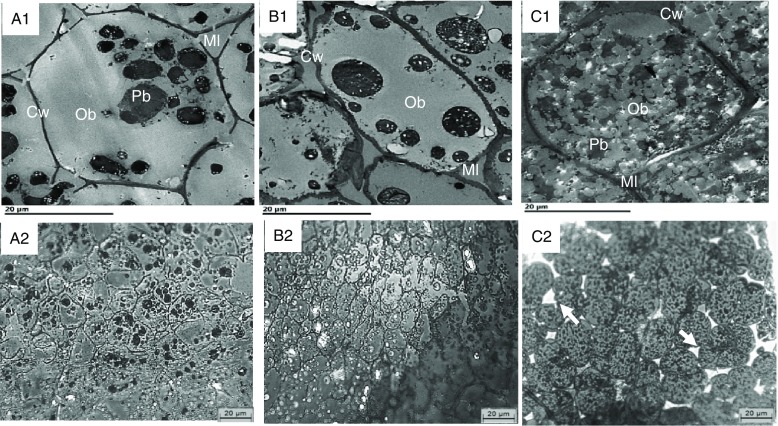
Transmission electron microscopy (TEM) (A1–C1) and light microscopy (LM) (A2–C2) of undigested walnuts (A1, A2), almonds (B1, B2) and pistachios (C1, C2) show intact cells and their contents. Cw, cell wall; Ml, middle lamella; Pb, protein body (black); OB, oil body (grey). Arrows point to loosely packed parenchyma cells. Scale bars = 20 μm.

The internal structure of cotyledons (e.g. lipid-bearing tissue) was observed in pre- and post-digested nuts using light as well as TEM. Undigested cotyledon consists of compactly packed isodiametric parenchymal cells, with an intact middle lamella (the zone defining the boundary between walls form adjacent cells), and intact (undamaged) cell walls. The sizes of the walnut, almond and pistachio cells were comparable (33, 31 and 35 µm, respectively). The raw walnuts had thin cell walls compared with the roasted almonds and pistachios (Table 3). Within undigested nuts, nutrients remained encapsulated within the cell (Fig. 4). As noted in prior reports^(31–34)^, intracellular OB were the most representative storage components. These lipids are protein-stabilised OB as their entire surface is covered by protein bodies or oleosins^(33,34)^. TEM micrographs showed variation in the organisation of lipid between nuts. In the raw walnut and roasted almond (Fig. 5(A1) and (B1), respectively), lipid consisted of a single and dense agglomerate, whereas in the roasted pistachio, lipid was organised into smaller dispersed droplets (Fig. 5(C1)). Furthermore, light imaging (40× objective) showed that parenchymal cells from raw walnuts and roasted almonds exhibited tightly packed cells (Fig. 5(A1) and (B1), respectively), whereas from roasted pistachios, cells were more loosely packed; this difference is probably caused by roasting, as reported by other investigators^(33)^ (Fig. 4 (C1)).

**Table 3.** Mean diameter of cell oil bodies (OB) and thickness of cell walls for undigested (raw) and post-digested nuts (Mean values with their standard errors; *n* 20)

	OB diameter per cell (µm)				Cell wall thickness per cell (µm)			
	Undigested		Post-digested		Undigested		Post-digested	
	Mean	sem	Mean	sem	Mean	sem	Mean	sem
Walnuts	28·54^a^	2·53	14·77^a,b^	2·92	0·862^a^	0·09	1·39^a^	0·14
Almonds	34·11^a^	2·53	21·07^b^	2·93	1·58^b^	0·09	1·48^a^	0·14
Pistachios	3·63^b^	2·53	8·80^a^	2·93	1·39^b^	0·09	1·38^a^	0·14

a,b Mean values in a column with unlike superscript letters are significantly different (*P* < 0·05; repeated-measures ANOVA).

**Fig. 6.**
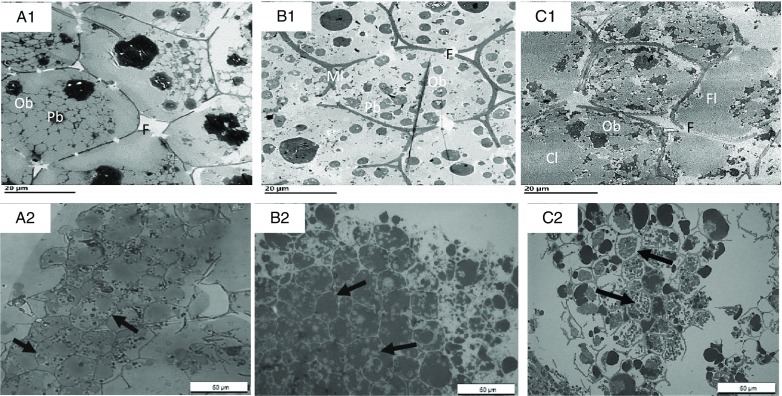
Transmission electron microscopy (TEM) (A1–C1) and light microscopy (LM) (A2–C2) micrographs of sections of nut tissues from walnuts (A1, A2), almonds (B1, B2) and pistachios (C1, C2) recovered after mastication. F, fissures; Ml, middle lamella; OB, oil body; Cl, coalesced lipid; Fl, free lipid. Arrows point to intact cells underneath the fractured layer of parenchyma tissue; note coalesced lipid (OB) from fractured cells and free lipid on the peripheral edge of the tissue. Scale bar A1–C2 = 20 µm; A2–C2 = 50 µm.

**Fig. 7.**
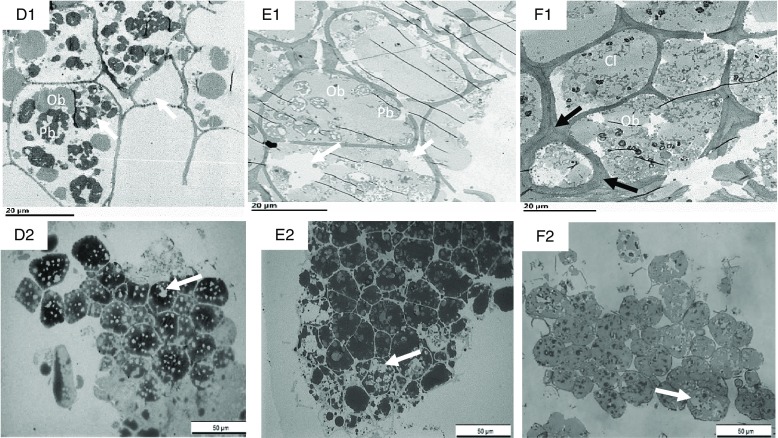
Transmission electron microscopy (TEM) (D1–F1) and light microscopy (LM) (D2–F2) micrographs of sections of nut tissues from walnuts (D1, D2), almonds (E1, E2) and pistachios (F1, F2) recovered after 60 min of the gastric phase. OB, oil body; Cl, coalesced lipid; Pb, protein body. Black arrows show thickened cell walls with thickened middle lamella at junction zone; white arrows show depletion of intracellular contents. Scale bar D1–F2 = 20 µm; D2–F2 = 50 µm.

**Fig. 8.**
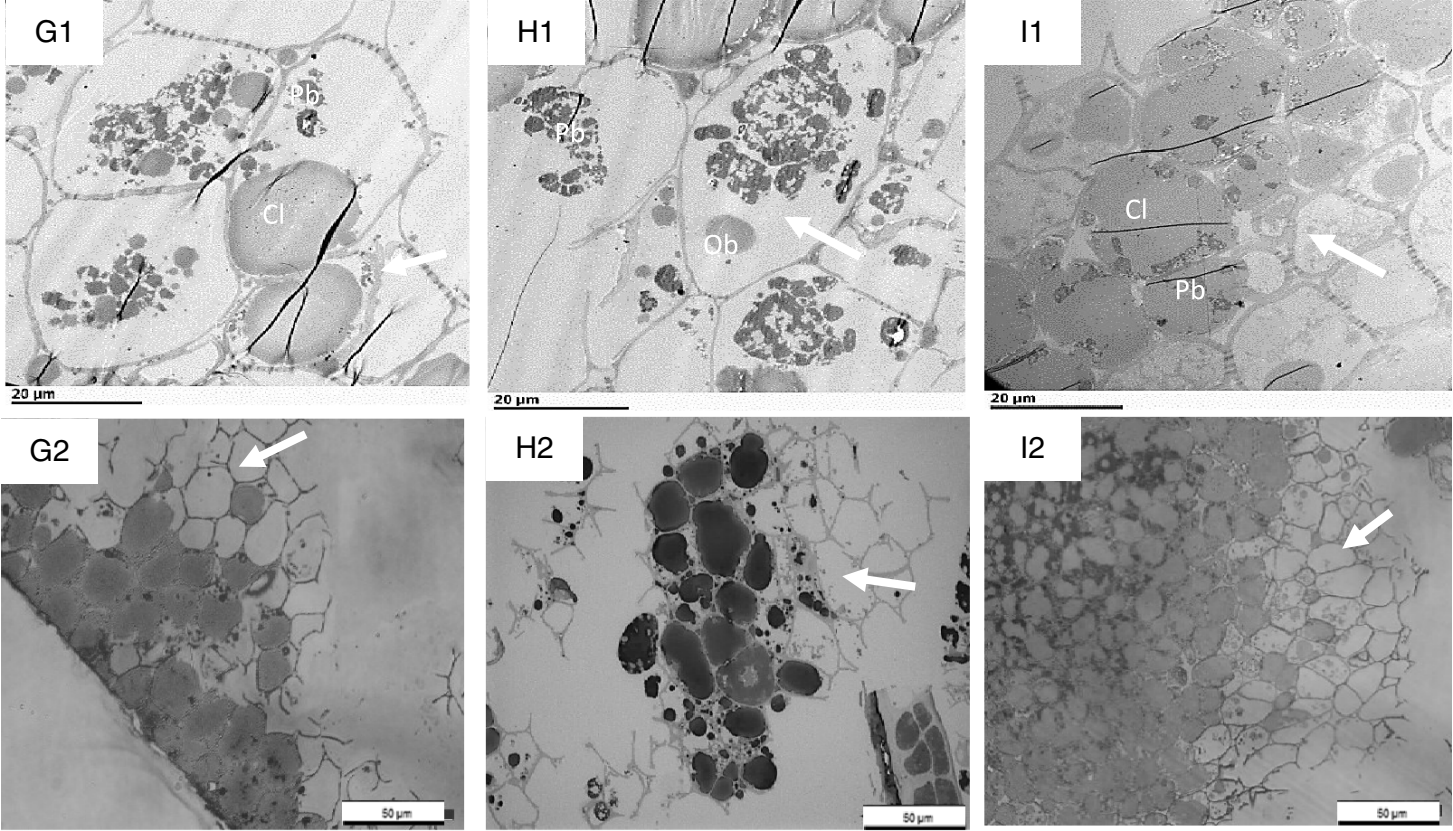
Transmission electron microscopy (TEM) (G1–I1) and light microscopy (LM) (G2–I2) micrographs of sections of nut tissues from walnuts (G1, G2), almonds (H1, H2) and pistachios (I1, I2) recovered after 120 min of the intestinal phase. Cl, coalesced lipid; OB, oil bodies; Pb, protein body. Arrows show depleted intact cells. Scale bar G1–I2 = 20 µm; G2–I2 = 50 µm.

Figs. 6–8 compare the micrographs of walnuts, almonds and pistachios after mastication and *in vitro* digestion. Following mastication, the cell walls for each nut appeared fissured. For walnuts, cell distortion and rupturing rather than separation were observed mainly in peripheral cells located beneath the fractured surface, increasing intracellular nutrient accessibility to digestive enzymes (Fig. 6(A1)). Moreover, portions of the OB were clearly organised into smaller spherical structures when compared against the undigested nut (Fig. 6(A1)). For almonds, the first layer of cells was largely ruptured, as in the walnuts, and released cellular contents (Fig. 6(B1)). A higher level of cellular integrity was observed in the underlying cells, which is consistent with previous microstructural studies with almonds^(8,12)^. Extensive cell wall degradation was observed in the pistachios compared with the almonds and walnuts (Fig. 6(C1)).

After gastric digestion, most protein bodies in walnuts appeared aggregated and disassociated from the surface of the OB, resulting in their coalescence (Fig. 7(D1)). In almonds, protein bodies remained attached to the oil droplet surfaces (Fig. 7(E1)), while in pistachios, proteins were mostly disrupted and in some cases, remnants of the protein bodies adhering to the lipid droplets could be found (Fig. 7(F1)). Post digested pistachios exhibited smaller OB compared with the walnuts and almonds (Table 3). Moreover, for pistachios, a thickened middle lamella was noted, possibly suggesting that some cell wall swelling may have occurred under gastric conditions (Fig. 7(F1)). However, careful interpretation of the data is required when examining nut tissues with electron microscopy. Further ruptured cell walls were identifiable in the roasted almonds, and the intracellular compounds are clearly accessible (Fig. 7(E1)). Some cell separation was seen in the walnuts probably due to the acidic hydrolysis of middle lamella reducing cell-cell adhesion^(13,19)^ (Fig. 7(D2)). Progressive degradation of lipid and intracellular contents was observed when tissues collected after chewing (Fig. 6(A1)–(C1), (A2)–(C2) were compared with tissues after gastric (Fig. 7(D1)–(F1), (D2)–(F2)) and intestinal digestion (Fig. 8(G1)–(I1), (G2)–(I2)). Further, undigested lipid and protein bodies were clearly visible following the intestinal phase in all nut samples.

## Discussion

Clinical trials document limited efficiency of energy extraction from almonds^(18)^, pecans^(35)^, peanuts^(9)^, pistachios^(16)^ and walnuts^(17)^. The most widely proposed mechanism for this effect highlights the structural integrity of cell walls and their encasement of energy-yielding nutrients (especially fat in the case of nuts). However, this mechanism does not account for the published energy losses from walnuts, almonds and pistachios. The former two reportedly yield about 80 % of their predicted energy (based on Atwater factors), while the measured yield from the latter was reported as approximately 95 %. The physical properties (i.e. hardness) of these three nuts would predict a rank ordering of: almonds > pistachios > walnuts (largest to smallest particle size). This suggests additional factors may be involved in the response to digestive processes for these nuts. Being less brittle, mastication of walnuts was expected to lead to cell separation more than cell wall fracturing, resulting in smaller particle sizes, but a higher proportion of intact cells leading to a low bioaccessiblity comparable to almonds. In contrast, based on the brittleness of almonds, their parenchymal cells were predicted to fracture, but because almonds require considerable masticatory effort, it was predicted that they would be swallowed as large particle sizes. It was also predicted that the greater digestibility of pistachios was due to higher cell wall degradation throughout the GI tract, leading to better energy bioaccessiblity. The present trial explored these hypotheses. In contrast to some previous studies concerning lipid bioaccessibilty of nut tissues^(11)^, the present design ensured that the role of oral processing was included in the analysis. Samples tested in *in vitro* gastric and intestinal models were chewed by humans under naturalistic conditions and drawn from the participants at the point they chose to swallow.

Not surprisingly, fewer chewing cycles were required to reach the swallowing threshold for walnuts and pistachios compared with almonds. The observed difference in chewing cycles may partially relate to their physical characteristics (e.g. hardness and brittleness)^(36)^. Increased food hardness is associated with a greater number of chews before swallowing^(37)^. Roasting nuts also result in smaller particles after mastication than oral processing of raw nuts^(25)^. The more malleable structure of walnut tissue could facilitate swallowing larger particles^(38)^. Additionally, the thinner cell walls (Table 3) and more disrupted parenchyma (Fig. 6) in the walnuts and pistachios, respectively, may have resulted in structures that were more easily fractured and hydrated by saliva during mastication. However, weak structure is not a likely explanation here as we previously demonstrated that under fixed chewing conditions, walnuts do not degrade to a greater degree than almonds or pistachios^(36)^. Since there were differences in chewing between the almonds (roasted) and the pistachios (dry-roasted) compared with the walnuts, there may also be an effect of roasting on masticatory behaviour. Such an effect has been reported^(39)^. Moreover, different ways of roasting (e.g. hot air *v*. oil roasting, variation in heating temperatures and times) lead to alterations in the number of chews mainly by changing the parenchyma structure and properties^(19)^. The larger particle size (volume mean diameter ([d_43_]) in the walnuts after simulated GI digestion is in line with previous reports^(26)^, indicating that GI conditions destabilise some of the walnut protein bodies (oleosins) that may have led to OB aggregation/coalescence. This aggregation/coalescence can exert pressure on the cell walls and thus the volume of the recovered particles. Walnut proteins are primarily glutelin, which are readily denatured by low pH, as would occur in the stomach^(26)^. In contrast, almonds showed a continuous decrease in mean particle size during 60 min of gastric and 120 min of intestinal digestion. This can be attributed to the erosion of almond particles or to their resistant interfacial proteins (amandin and other almond proteins) to hydrolysis by pepsin. This could result in higher OB stability and less aggregated proteins^(40)^. Thus, there is greater surface area for digestive enzymes and bile to access. The change in particle size of almonds is in agreement with that reported for raw, sliced almonds and roasted hazelnut OB preparations^(15,40)^. For pistachios, the small d_43_ values after gastric digestion might reflect an enhanced stability against OB aggregation. Hydrophilic components of its protein bodies are hydrolysed during roasting rendering them more lipophilic and better suited to stabilise the OB^(15)^. Conversely, roasting of pistachios could have accelerated the disintegration of particles during *in vitro* gastric digestion, as has been demonstrated with roasted almonds and roasted peanuts^(21,22)^. However, OB in roasted nut (almond) cells tend to coalesce during digestion^(12)^, likely due to the development of more porous or fractured cell walls from the heating process. This allows cellular infiltration of the digestive juices and consequent destabilisation of the OB. This could explain the higher d_43_ values of the pistachios after intestinal digestion. This is in agreement with a previous study for almond extract (free OB), where the natural layer surrounding the almond OB induced a stronger decrease in TAG absorption and appearance in the blood postprandially compared with almond oil emulsions^(41)^.

It has been shown that trituration of almonds by oral or mechanical processing increases the release of lipid from the cells on the periphery of particles as a result of cell rupture^(8)^. Because of the different physical properties (i.e. soft texture) of walnut seeds, we predicted that chewing would result in cell separation rather than fracture with reduced release of lipid from walnut tissue. Contrary to this expectation, walnut cells ruptured, rather than separated which is probably due to their strong cell–cell adhesion (Fig. 5(A2)). In nuts and seeds, cell separation is caused mainly by weakening the cell–cell adhesions during gastric digestion, as can be seen in the raw walnuts (Fig. 6(D1)). However, we observed that nut cells have the potential to separate because of thermal processing, which can be seen in the micrograph of the undigested pistachios (Fig. 5(C2)). No studies performed so far have shown any evidence of cell separation occurring in raw or even thermally processed nuts that have been chopped, or chewed, except in ingested nuts after gastric digestion *in vitro* or microbial fermentation *in vivo* ^(8,13)^. Our findings indicate that tissue fracturing rather than cell separation may be the main mode of tissue failure in walnuts.

Grundy *et al.* recently reported a negative linear relationship (*R* ^2^ 0·65) between particle size and NEFA release for both raw and roasted almonds^(42)^. Based on these data, it may be expected that the smaller particle sizes would exhibit greater nutrient losses. This corresponded to the sample with the largest proportion of ruptured cells on the surfaces of the particles. The lipid released from these fractured cells would be more accessible to intestinal lipase. However, our results show that the amount of lipid released is not a function of the number of ruptured cells on the fractured surface of walnut tissue. These observations are consistent with previous studies that also demonstrated non-linear relationships between particle size and nutrient bioaccessibility^(30,43)^. For example, a study with raw and cooked carrots (gently and intensely cooked) showed that the dependency of *β*-carotene bioaccessibilty on particle size became more pronounced as the thermal process became less intense^(44)^. Previous studies from our group also showed that increasing the intensity of mastication resulted in a higher lipid release from almond tissues, but no specific dependency of the lipid bioaccessibility on particle size was observed^(43)^.

The loss of lipid from particle surface cells suggests that the cell wall becomes a less efficient barrier to digestion with time^(45)^. There is now convincing evidence that the internal structure of nuts (oil droplets, protein bodies) can be retained to a greater or lesser extent during digestion and can variably hinder or augment digestion and absorption. As a result, we suggest that the structural integrity (intact cells) may not be the primary factor in influencing lipid bioaccessibility in walnuts and other nuts and that the internal structure of the nut content has the potential to greatly influence postprandial lipid metabolism. Digestion of OB has been studied in almonds, walnuts and hazelnuts^(15,26,40)^. These studies show that gastric digestion of oleosins allows more rapid access of the lipase to the oil–water interface for efficient lipolysis of the OB. Interestingly, *in vitro* intestinal digestion of a walnut OB showed the spontaneous formation of a multiple emulsion. This was probably driven by the interaction of PUFA as NEFA and 2-monoacylglycerol, walnut peptides from proteolysis by digestive enzymes and negatively charged bile salts^(26)^. The oil and water droplets were stabilised by crystals of lipolytic products and/or bile salts, and these structures are predicted to play a major role in how lipids are digested and absorbed from walnuts. These results confirm that in addition to intact cells, there are other physiochemical factors, such as the nature of the interfacial layer, that influence the extraction of energy from walnuts.

An additional factor influencing lipid bioaccessibility may be the increase in porosity of the cell wall during digestion because of swelling of the cell walls during digestion. This would increase the influx of lipase and subsequent leakage of hydrolysed lipids. Some evidence of cell wall swelling has been reported for raw and roasted nuts^(21,22)^. In the present trial, an increase in porosity may have occurred especially for walnuts (Table 3) and pistachios (Fig. 5(F1)). However, in almonds, the swelling of cell walls has been previously shown to occur slowly and over much longer times (i.e. 3–24 h)^(11,21,22)^.

In our *in vitro* digestion experiment, the quantity of non-digestible lipid was higher (~22 %) for pistachios than that has been previously reported in human studies (~5 %)^(16)^. This may reflect differences in roasting conditions (e.g. temperature and time). Roasting induces microstructural (e.g. lipid coalescence) and chemical changes (e.g. partial cell wall rupture, cell wall swelling and protein denaturation) that facilitate lipolysis^(46)^.

The fraction of lipid was comparable for walnuts and almonds (i.e. 24 %) after the intestinal phase of digestion. These values are markedly lower that those reported previously which indicated as much as 47 % of the lipid remained in the cellular structure of almond tissue at this stage of digestion. Given findings of lipid excretion in the range of 21 and 24 % for walnuts and almonds, respectively, from *in vivo* studies^(17,18)^, the previous data require that a high proportion of lipid is extracted in the colon. The effects of lipid reaching the colon either undigested or in digested form and its interactions with the gut microbiota are unclear. Emerging research indicates that both the type and form of nuts may differentially alter microbial metabolism in the colon^(6,47)^. The present values are in line with little lipid loss in the colon. Future studies in this area are required to determine the role of the gut microbiota in lipid metabolism. It is also possible that the discrepancy in the previous and present studies in lipid bioaccessibility estimates only reflects methodological approaches. Different amounts of shaking to mimic the mixing/force in the gastric phase of digestion, types/concentrations of enzymes introduced, different digestion conditions/times and/or a difference in the amount of material digested could potentially explain the observed difference^(25,48,49)^.

Our study has limitations. One objective of the study was to quantify the number of cells from walnuts that remain intact during digestion compared with almonds and pistachios. Our data were derived from two-dimensional images and manual calculations, so careful interpretation of the data is required. Future study should consider measuring cell size for the calculation of the proportion of fractured cells. Also, large particles were excluded in the particle size analysis, which may show some relationship to bioaccessiblity not captured by our method of measuring particle size. It should also be emphasised that the nuts assessed were tested in forms commonly consumed rather than as a common form of processing and the latter may influence microstructure and lipid coalescence. Our study also has strengths. To the best of our knowledge, this is the first study to contrast the effects of oral, gastric and intestinal processing on the cellular degradation between different nut types. Another strength is that the results provide more information concerning the mechanisms that account for the inefficiencies in extracting energy from walnuts and other nuts.

### Conclusion

Nut structure and internal constituent properties may decrease lipid bioaccessibilty during digestion. Understanding the mechanisms that allow nuts to be a highly energy dense food without promoting positive energy balance is of particular intest since nuts are an increasingly consumed food with postive health benefits and new strategies could be developed to optimise nut-based functional ingredients. Our results show that chewing causes a rupture of cell walls but the amount of lipid released does not correspond with the number of ruptured cells on the fracture surface of nut tissue. Moreover, the ratio of ruptured cells to intact cells was not related to particle size. In this work, evidence of additional mechanisms by which the structural features of nuts can reduce lipid bioaccessibility was provided. Examination of nut microstructure indicates that the fissures of cell walls as well as lipid storage properties are also important for energy extraction. These findings indicate walnuts, almonds and pistachios yield similar, but limited amounts of energy (~80 %) during digestion, likely through varied mechanisms. For nuts, including walnuts, the limited bioaccessibility may stem in part from the ready hydrolysis of their oliosins at low pH allowing for OB coalescense and resistance to lipolysis.
